# Clinical Relevance of Transorbital Ultrasonographic Measurement of Optic Nerve Sheath Diameter (ONSD) for Estimation of Intracranial Pressure Following Cerebrospinal Fluid Diversion Surgery

**DOI:** 10.7759/cureus.25200

**Published:** 2022-05-22

**Authors:** Akhilendra Chopra, Praveen K Das, Samiksha Parashar, Shilpi Misra, Manoj Tripathi, Deepak Malviya, Deepak Singh

**Affiliations:** 1 Anaesthesiology and Critical Care, Dr. Ram Manohar Lohia Institute of Medical Sciences, Lucknow, IND; 2 Neurosurgery, Dr. Ram Manohar Lohia Institute of Medical Sciences, Lucknow, IND

**Keywords:** intracranial pressure, optic nerve sheath diameter, ultrasound, ventriculo-peritoneal shunt, csf diversion procedures

## Abstract

Background and aim

Raised intracranial pressure (ICP) can be estimated by various invasive as well as non-invasive techniques. Optic nerve sheath diameter (ONSD ) is a bedside non-invasive technique for assessment of ICP as a regular follow-up tool and has added advantage over CT scan/MRI, which require patient transfer to the suite. Cerebrospinal fluid (CSF) diversion procedures such as a ventriculoperitoneal shunt or external ventricular drainage are commonly done to relieve symptoms of patients with raised ICP. Change in ICP measured through ONSD after CSF diversion procedures may guide the proper functioning of the shunt and immediate post-operative management. The present study was conducted to compare ONSD before and after CSF diversion procedures and correlate the ONSD with ICP. Our secondary objective was to determine the ONSD cutoff for the prediction of ICP >20mm Hg.

Setting, design, and methods

This prospective, comparative, and observational study was carried out at Dr. Ram Manohar Lohia Institute of Medical Sciences, Lucknow, India. The present study was conducted on 40 adult patients undergoing CSF diversion surgery under general anaesthesia. Ultrasonographic measurement of the ONSD was performed before induction, after induction, after endotracheal intubation, after completion of shunt surgery, and then every two hours for 12 hours. The direct ICP was measured by the neurosurgeon at the time of the initial ventricular puncture.

Statistical analysis

The Wilcoxon signed-rank test was used to compare pre and post variables. Qualitative variables were compared using the Chi-Square test/Fisher’s exact test as appropriate. Spearman's rho statistical measure of linear association was applied to measure the strength of linear association between parameters to show how close the points lie to a straight line. A p-value of <0.05 was considered statistically significant.

Results

The mean value of ONSD before induction and after induction was 6.36 ± 0.61 mm and 6.29 ±0.64 mm, respectively. After endotracheal intubation, ONSD slightly increased to 6.34 ±0.62mm, followed by a consistent decrease in ONSD values. The mean direct ICP recorded was 30.93±6.22 mmHg. Comparison of mean ONSD before induction, after induction, and after intubation with ONSD after surgery was statistically significant (p <0.001). We found a strong positive correlation between direct ICP and ONSD after intubation with a correlation coefficient of 0.969 (P <0.001). Receiver operating characteristic (ROC) curve analysis showed an ONSD cutoff of >5.85, predicted ICP>20 mmHg with a sensitivity of 92.3%, and specificity of 85.7%.

Conclusion

Measurement of ONSD by ultrasonography is an important and reliable tool in the assessment of normalization of ICP post CSF diversion procedure.

## Introduction

Hydrocephalus is a disturbance of cerebrospinal ﬂuid (CSF) production, ﬂow, or absorption, leading to elevation in intracranial pressure (ICP). CSF diversion procedures like ventriculoperitoneal shunt or external ventricular drainage are commonly done in patients with hydrocephalus, to relieve the raised ICP when immediate definitive management is not possible [1]. Assessment of the ICP after these procedures can guide proper post-operative management. Changes in the ICP can be assessed clinically, radiologically, or invasively by the intraventricular catheter. Clinical assessment depends on the subjective assessment of the clinician, whereas for radiological assessment patient needs to be shifted to the radiological suite. Also, inserting a catheter in intra-ventricular space for the sole purpose of ICP measurement is invasive.

Increased ICP is transmitted to the subarachnoid space surrounding the optic nerve, due to which expansion of optic nerve sheath diameter (ONSD) occurs. Though ventriculostomy is considered the gold standard method for accurate measurement of ICP, micro transducers measure just as accurate value [2]. ONSD measurement being a safe point of care and reliable technique has emerged as a useful method to assess the ICP [3].

A significant reduction in ONSD following endoscopic third ventriculostomy or brain tumour removal has been reported in children with hydrocephalus [4] but no such study has been done in the adult population. Also, the time scale of change in the reduction of ICP is uncertain. So, we hypothesize that change in ONSD detected through trans-orbital ultrasonography could help ascertain the change in ICP following CSF diversion surgery.

Our primary objective was to compare and correlate the ONSD with ICP following ventriculoperitoneal shunt surgery. Our secondary objective was to determine the ONSD cutoff for the prediction of ICP >20mm Hg.

## Materials and methods

After getting approval from the institutional ethical committee of Dr. Ram Manohar Lohia Institute of Medical Sciences, Lucknow, Uttar Pradesh, India (IEC No-80/17), this prospective, comparative, observational study was conducted at Dr. Ram Manohar Lohia Institute of Medical Sciences, Lucknow, Uttar Pradesh India. Prior to the enrollment of patients, written informed consent was obtained from all the patients. The study spanned a duration of 18 months. Strengthening the Reporting of Observational Studies in Epidemiology (STROBE) guidelines were followed and implicated in this prospective observational cohort study [5,6].

Patients aged between 18 to 65 years of either sex, American Society of Anesthesiologist (ASA) physical status 1-3, undergoing CSF diversion procedure (ventriculoperitoneal shunt surgery under general anaesthesia) or revision shunt surgery, were included in the study. Patients with anatomical or functional abnormalities of the optic nerve or vision loss due to optic atrophy were excluded from the study.

All patients underwent the standard institutional protocol of overnight fasting, premedication, and institution of general anaesthesia. Baseline parameters including pulse rate, non-invasive blood pressure (NIBP), oxygen saturation (SpO2), and ECG were monitored throughout. After endotracheal intubation, mechanical ventilation was started with a tidal volume of 8 ml/kg, and the respiratory rate was adjusted to maintain EtCO2 of 35 ± 2 mm Hg during surgery. Temperature was maintained between 35.5°C to 37°C by using a nasopharyngeal temperature probe. Anaesthesia was maintained with 50% oxygen/air and isoflurane concentration between 0.8 to 1.2 MAC during the optic nerve sheath diameter measurements.

CSF diversion surgery was performed in a standardised manner using Kocher’s point entry for the proximal catheter and paraumbilical entry for the distal catheter [7]. A programmable ventriculoperitoneal shunt system was inserted, with a one-way check valve that opens and drains the CSF when the intra-ventricular pressure rises above a speciﬁc pressure. The direct ICP was measured by the neurosurgeon at the time of the initial ventricular puncture.

A single investigator, trained in transorbital ultrasonography, took all the ONSD measurements, using the M-turbo ultrasound image system. The patient was placed in a neutral supine position on the operating table and ultrasound gel was applied over the closed upper eyelid. A linear 13to 6-MHz ultrasound probe was placed on the lateral area of the eyelid, avoiding excessive pressure on the eye. The transverse diameter of the ONSD 3 mm behind the globe was measured using an electronic calliper. An average of three measurements were done for each eye at each time point and the mean of these values was considered ONSD of that eye.

Ultrasonographic measurement of the ONSD was performed before induction of anaesthesia, after induction of anaesthesia, after endotracheal intubation, after completion of shunt surgery and then two-hourly for 12 hours. The direct ICP measured by the neurosurgeon at the initial ventricular puncture was also recorded. The systolic blood pressure, diastolic blood pressure, and heart rate were recorded throughout the study. 

Statistical analysis 

A review of the literature suggested that the mean difference in the preoperative and postoperative ONSD of patients with raised ICP and undergoing CSF diversion procedures is normally distributed with a standard deviation of 2mm [8]. A sample size of 40 study subjects was calculated, to be able to reject the null hypothesis (that the mean difference is 0 mm) with a power of 80%. Type 1 error probability of this test of null hypothesis is 0.05.

All the data were collected and recorded electronically by a single observer. Data from the patients with failed shunt surgery were not analyzed. Failure of the shunt operation was deﬁned by the neurosurgeon, on the ﬁrst or second day after surgery, according to the patient’s radiological ﬁndings and clinical symptoms.

Categorical variables were presented in number and percentage (%) and continuous variables were presented as mean ± SD. Paired t-test was used to compare pre and post variables for scale parameters. Qualitative variables were compared using the Chi-Square test/Fisher’s exact test as appropriate. A p-value of <0.05 was considered statistically significant. The data were entered in an Excel spreadsheet (Microsoft Corporation, Redmond, Washington, United States) and analysis was done using SPSS for Windows, Version 16.0 (Released 2007; SPSS Inc., Chicago, United States).

## Results

A total of 40 patients were included in the study and surgery was successful in all 40 patients. Patients were aged between 18 years to 59 years with a mean age of 35.50 years. Of the patients, 47.5% were male and 52.5% were female. The most common etiological factor for hydrocephalus was space-occupying lesions, comprising 50% of cases, while tuberculous meningitis (TBM) with communicating hydrocephalus comprised 25% of cases. Table 1 depicts the etiological distribution of hydrocephalus. Majority of shunts inserted for CSF diversion procedure were right side medium pressure ventriculo-peritoneal (MPVP) shunt (55%), while right LPVP, left MPVP, left LPVP were 15%, 15%, 12.5%, respectively, and in one case (2.5%) endoscopic third ventriculostomy (ETV) procedure was used for drainage of CSF.

**Table 1:** Etiological distribution of hydrocephalus TBM: tuberculous meningitis; GTCS: generalized tonic-clonic seizure

Etiology	N	Percent
Aqueductal stenosis hydrocephalous	1	2.5
Space occupying lesion	23	57.5
Left supraclinoid aneurysm	1	2.5
Neurocysticercosis with GTCS	1	2.5
Post traumatic hydrocephalous	1	2.5
Revision of shunt	2	5.0
TBM with communicating hydrocephalous	10	25.0
TBM with noncommunicating hydrocephalous	1	2.5
Total	40	100.0

Direct ICP and ONSD at different time intervals are shown in Table 2. We observed that after anaesthesia induction there was a decrease in ONSD. The mean value of ONSD before induction and after induction was 6.36 ± 0.61 mm and 6.29 ± 0.64 mm, respectively, while after endotracheal intubation ONSD slightly increased compared to induction value, measured as 6.34 ± 0.62mm, followed by a consistent decrease in ONSD values. The mean direct ICP recorded was 30.93±6.22 mmHg. 

**Table 2:** Measured values of Direct ICP and ONSD at different time intervals ICP: intracranial pressure; ONSD: optic nerve sheath diameter; N: number of patients

	N	Minimum	Maximum	Mean	Std. Deviation
Direct ICP (mm Hg)	40	18.00	44.00	30.93	6.22
ONSD before induction	40	5.35	7.85	6.3575	0.61201
ONSD after induction	40	5.30	7.90	6.2937	0.64283
ONSD after intubation	40	5.35	7.85	6.3362	0.62879
ONSD after surgery	40	4.85	5.70	5.2588	0.21688
At 2 hours	40	4.05	5.50	5.0762	0.26067
At 4 hours	40	4.55	5.55	5.0125	0.21506
At 6 hours	40	4.45	5.55	4.9475	0.23341
At 8 hours	40	4.45	5.40	4.9000	0.23122
At 10 hours	40	4.30	5.30	4.8438	0.24863
At 12 hours	40	4.40	5.25	4.8075	0.23303

Comparison of mean ONSD before induction, after induction, and after intubation with ONSD after surgery (Table 3) was statistically significant (p-value <0.001). We found in our study that ONSD was signiﬁcantly reduced after CSF diversion procedures.

**Table 3:** Mean comparison of ONSD before induction, after induction, after intubation with ONSD after surgery ONSD: optic nerve sheath diameter

	Mean	N	SD	p-value
ONSD before induction	6.36	40	0.61201	<0.001
ONSD after surgery	5.26	40	0.21688	
ONSD after induction	6.29	40	0.64283	<0.001
ONSD after surgery	5.26	40	0.21688	
ONSD after intubation	6.34	40	0.62879	<0.001
ONSD after surgery	5.26	40	0.21688	

**Figure 1:**
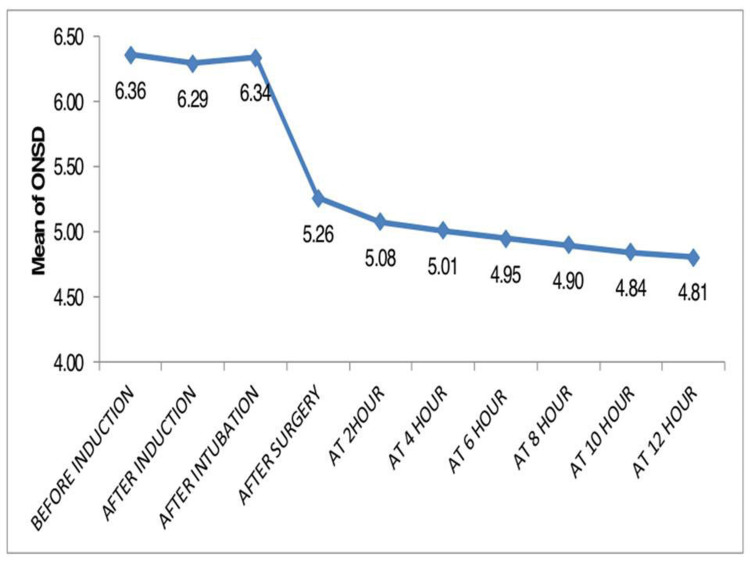
ONSD before and after CSF diversion procedure: shows that ONSD comes near to 5 mm at around five hours post-CSF diversion procedure ONSD: optic nerve sheath diameter; CSF: cerebrospinal fluid

**Figure 2:**
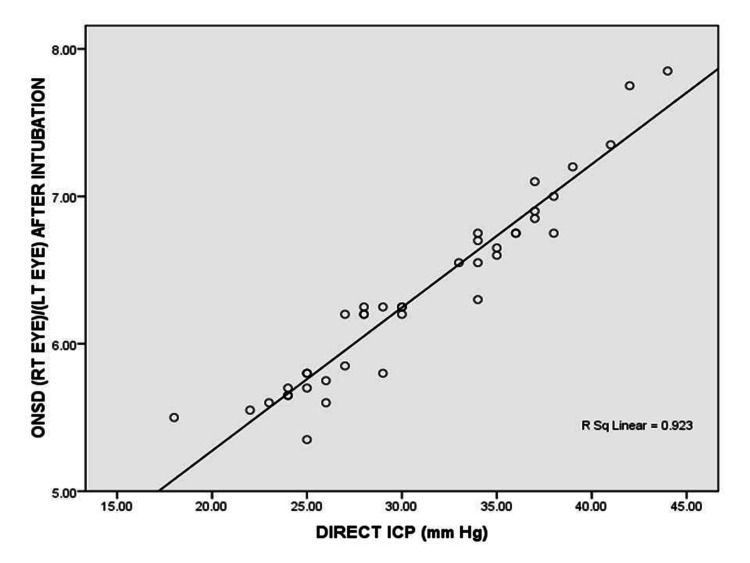
Correlation of direct ICP with mean ONSD after intubation ICP: intracranial pressure; ONSD: optic nerve sheath diameter

**Figure 3:**
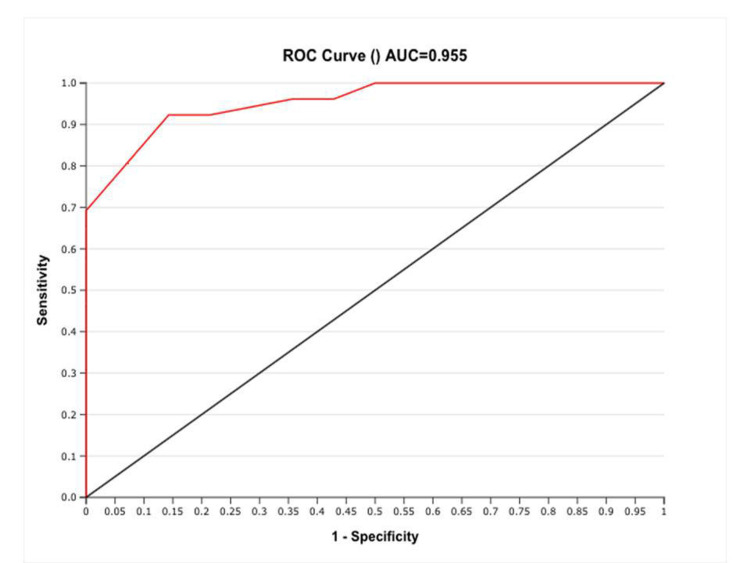
ROC curve analysis for ONSD cutoff to predict ICP >20 mmHg ROC: receiver operator characteristic; ONSD: optic nerve sheath diameter; ICP: intracranial pressure; AUC: area under the curve

Observing the serial values of ONSD, showed that ONSD comes near to 5 mm at around five hours post CSF diversion procedure.(Figure 1). We found a strong positive correlation between direct ICP and ONSD after intubation with correlation coefficient of 0.969 and which was statistically highly significant with P-value <0.001. Figure 2 shows the scatter plot between Direct ICP and ONSD after endotracheal intubation. ROC curve analysis showed that the ONSD cutoff of >5.85 mm predicted ICP >20 mmHg with a sensitivity of 92.3% and specificity of 85.7%. Figure 3 shows the best cutoff value and area under the ROC curve for the prediction of ICP >20 mmHg.

## Discussion

A total of 40 patients were included in the study. After the surgery, till 12 hours no shunt malfunction was detected in any patient. We found that there was a significant and continuous reduction in ONSD after the surgery when compared to baseline. ONSD positively and significantly correlated with direct ICP and an ONSD cutoff of >5.85 mm predicted ICP>20mm Hg.

Trans-orbital ultrasonography-guided ONSD measurement has emerged as a safe, point of care and reliable technique to assess the ICP noninvasively. Change in ONSD could help ascertain the change in ICP following CSF diversion surgery and if ICP does not fall after these diversion procedures, it can be emphatically diagnosed and an immediate management plan can be undertaken. The optic nerve sheath is the perineural compartment around the optic nerve filled with CSF, with direct communication with the craniospinal subarachnoid space. Thus, the ONSD can reflect intracranial hypertension immediately [9].

We measured the transverse diameter of the ONSD 3 mm behind the globe using an electronic calliper. This method has been suggested as reliable,reproducible as well as correlating [10], as maximum ONSD fluctuations has been noted in the dura mater at 3 mm behind the papilla [10]. Various studies have concluded that ultrasonic measurement of ONSD shows a good level of diagnostic accuracy for detecting intracranial hypertension and in clinical decision-making [3,11-16].

We observed that the ONSD signiﬁcantly and continuously reduced after the CSF diversion procedure. The mean value of ONSD before induction was 6.36 ± 0.61 mm while mean value of ONSD after CSF diversion procedure was 5.2 ± 0.21 mm (p <0.001). Choi et al. [8] in their study also observed that the ONSD of children with hydrocephalus reduced immediately and significantly after they underwent ventriculoperitoneal shunt surgery concluding that the change in ONSD reflected the change in ICP, which was found in our study too. The rapidity of this reduction of ONSD, suggested that elasticity of the ONSD is preserved even in patients with long-standing raised ICP. Similar to our study, Hansen et al. concluded that elasticity of the anterior optic nerve sheath is sufficient for the detection of change in ONSD for upward pressure as well downward change [17]. Moretti et al also found that after one minute of CSF drainage, there was significant and rapid reduction in ONSD from 5.89±0.61 mm to 5.0±0.33 mm (p-value <0.01) [16]. They had concluded that ONSD is a reliable method to detect raised ICP.

On comparing direct ICP with ONSD after intubation, we observed a statistically significant and direct strong positive correlation (r>0.9, p<0.001)) between direct ICP and ONSD. The change in ONSD reflected the change in ICP. Linear correlation between ONSD and ICP has been found in various studies. A retrospective study evaluating the correlation between ONSD measured on pre operative brain CT and ICP, found that ONSD is linearly correlated with ICP (r = 0.543, P < 0.001) [18]. Another prospective observational study concluded that bedside ONSD by ultrasonography shows a strong correlation (r=0.59, p<0.0005) with direct ICP measured by invasive intracranial monitors [19]. This provides support for the use of ONSD as a noninvasive mode of investigation for elevated ICP.

We found a cutoff ONSD value of >5.85mm for prediction of ICP>20% with sensitivity of 92.3% and specificity of 85.7%. The cutoff value for raised ICP value varies widely in the literature. This variation could be attributed to the varying modes of ICP and ONSD measurement. Leea et al. found a cutoff value for ONSD 5.3 mm for raised ICP (88% sensitivity, 79% specificity) [12]. They obtained ONSD values from brain CT images. Tayal et al. reported that the ultrasonographically obtained ONSD cut-off value of 5 mm is 100% sensitive and 63% specific for the identification of raised ICP confirmed by CT [20]. Our study postulated that ONSD comes to a value near to 5 mm at around five hours post-CSF diversion procedure. So, we can make an inference that ICP comes to normal at about five hours post-surgery. To our knowledge, this is the first study that attempts to correlate the relationship between direct ICP and ONSD as a surrogate to ICP in adult patients undergoing CSF diversion surgery.

Limitations

There are some limitations to our study. First, the study is limited by its small size and the results should be validated in larger trials. Second, although, we used the standard technique for measurement of ONSD by ultrasonography, there may be other ways to measure ONSD more accurately, which is an area of ongoing research. Third, the direct ICP was only measured once, after the ventricle puncture, and not after drainage. The post‐drainage ICP was maintained under the specified valve pressure. Consequently, we were unable to assess the relationship between direct ICP and ONSD. However, we did maintain stable levels of anaesthesia, arterial pressure, heart rate, and end‐tidal carbon dioxide partial pressure throughout the surgery. Fourth, in our study, there was no incidence of shunt malfunction in the postoperative period, so evaluation of ONSD as a marker of malfunction of the shunt could not be assessed.

## Conclusions

To conclude, measurement of ONSD by ultrasonography is an important and reliable tool in the assessment of normalization of ICP, which may take around five hours to normalize post-CSF diversion procedure.We postulate that ONSD >5.85 mm as assessed by ultrasonographic measurement is a very reliable tool to diagnose raised ICP.

## References

[1] Singhal A, Yang MM, Sargent MA, Cochrane DD (2013). Does optic nerve sheath diameter on MRI decrease with clinically improved pediatric hydrocephalus?. Childs Nerv Syst.

[2] Raboel PH, Bartek J Jr, Andresen M, Bellander BM, Romner B (2012). Intracranial Pressure Monitoring: Invasive versus Non-Invasive Methods-A Review. Crit Care Res Pract.

[3] Major R, Girling S, Boyle A (2011). Ultrasound measurement of optic nerve sheath diameter in patients with a clinical suspicion of raised intracranial pressure. Emerg Med J.

[4] Goeser CD, McLeary MS, Young LW (1998). Diagnostic imaging of ventriculoperitoneal shunt malfunctions and complications. Radiographics.

[5] von Elm E, Altman DG, Egger M, Pocock SJ, Gøtzsche PC, Vandenbroucke JP (2007). The strengthening the reporting of observational studies in epidemiology (STROBE) statement: guidelines for reporting observational studies. PLoS Med.

[6] Eisenach JC, Kheterpal S, Houle TT (2016). Reporting of observational research in anesthesiology: the importance of the analysis plan. Anesthesiology.

[7] Winn HR, Youmans JR (2011). Youmans. Neurological Surgery, 6th edn. Youmans. Neurological Surgery, 6th edn.

[8] Choi SH, Min KT, Park EK, Kim MS, Jung JH, Kim H (2015). Ultrasonography of the optic nerve sheath to assess intracranial pressure changes after ventriculo-peritoneal shunt surgery in children with hydrocephalus: a prospective observational study. Anaesthesia.

[9] Hayreh SS (1964). Pathogenesis of oedema of the optic disc (papilloedema). A preliminary report. Br J Ophthalmol.

[10] Helmke K, Hansen HC (1996). Fundamentals of transorbital sonographic evaluation of optic nerve sheath expansion under intracranial hypertension. I. Experimental study. Pediatr Radiol.

[11] Shah SB, Bhargava AK, Choudhury I (2015). Noninvasive intracranial pressure monitoring via optic nerve sheath diameter for robotic surgery in steep Trendelenburg position. Saudi J Anaesth.

[12] Lee HC, Lee WJ, Dho YS, Cho WS, Kim YH, Park HP (2018). Optic nerve sheath diameter based on preoperative brain computed tomography and intracranial pressure are positively correlated in adults with hydrocephalus. Clin Neurol Neurosurg.

[13] Girisgin AS, Kalkan E, Kocak S, Cander B, Gul M, Semiz M (2007). The role of optic nerve ultrasonography in the diagnosis of elevated intracranial pressure. Emerg Med J.

[14] Blaivas M, Theodoro D, Sierzenski PR (2003). Elevated intracranial pressure detected by bedside emergency ultrasonography of the optic nerve sheath. Acad Emerg Med.

[15] Amini A, Eghtesadi R, Feizi AM (2013). Sonographic optic nerve sheath diameter as a screening tool for detection of elevated intracranial pressure. Emerg (Tehran).

[16] Moretti R, Pizzi B, Cassini F, Vivaldi N (2009). Reliability of optic nerve ultrasound for the evaluation of patients with spontaneous intracranial hemorrhage. Neurocrit Care.

[17] Hansen HC, Helmke K (1997). Validation of the optic nerve sheath response to changing cerebrospinal fluid pressure: ultrasound findings during intrathecal infusion tests. J Neurosurg.

[18] Chin JH, Seo H, Lee EH, Lee J, Hong JH, Hwang JH, Kim YK (2015). Sonographic optic nerve sheath diameter as a surrogate measure for intracranial pressure in anesthetized patients in the Trendelenburg position. BMC Anesthesiol.

[19] Kimberly HH, Shah S, Marill K, Noble V (2008). Correlation of optic nerve sheath diameter with direct measurement of intracranial pressure. Acad Emerg Med.

[20] Tayal VS, Neulander M, Norton HJ, Foster T, Saunders T, Blaivas M (2007). Emergency department sonographic measurement of optic nerve sheath diameter to detect findings of increased intracranial pressure in adult head injury patients. Ann Emerg Med.

